# Effects of Anthocyanin on Intestinal Health: A Systematic Review

**DOI:** 10.3390/nu13041331

**Published:** 2021-04-17

**Authors:** Thaísa Agrizzi Verediano, Hércia Stampini Duarte Martino, Maria Cristina Dias Paes, Elad Tako

**Affiliations:** 1 Department of Nutrition and Health, Universidade Federal de Viçosa, Viçosa 36570-000, MG, Brazil; thaisa.agrizzi@gmail.com (T.A.V.); hercia72@gmail.com (H.S.D.M.); 2 Empresa Brasileira de Pesquisa e Agropecuária (EMBRAPA), Sete Lagoas 35701-970, MG, Brazil; cristina.paes@embrapa.br; 3 Department of Food Science, Cornell University, Stocking Hall, Ithaca, NY 14850, USA

**Keywords:** microbiota, polyphenols, short chain fatty acids, intestinal barrier

## Abstract

Intestinal health relies on the association between the mucosal immune system, intestinal barrier and gut microbiota. Bioactive components that affect the gut microbiota composition, epithelial physical barrier and intestinal morphology were previously studied. The current systematic review evaluated evidence of anthocyanin effects and the ability to improve gut microbiota composition, their metabolites and parameters of the physical barrier; this was conducted in order to answer the question: “Does food source or extract of anthocyanin promote changes on intestinal parameters?”. The data analysis was conducted following the PRISMA guidelines with the search performed at PubMed, Cochrane and Scopus databases for experimental studies, and the risk of bias was assessed by the SYRCLE tool. Twenty-seven studies performed in animal models were included, and evaluated for limitations in heterogeneity, methodologies, absence of information regarding allocation process and investigators’ blinding. The data were analyzed, and the anthocyanin supplementation demonstrated positive effects on intestinal health. The main results identified were an increase of *Bacteroidetes* and a decrease of *Firmicutes*, an increase of short chain fatty acids production, a decrease of intestinal pH and intestinal permeability, an increase of the number of goblet cells and tight junction proteins and villi improvement in length or height. Thus, the anthocyanin supplementation has a potential effect to improve the intestinal health. PROSPERO (CRD42020204835).

## 1. Introduction

A healthy gut includes multiple positive aspects of the gastrointestinal (GI) tract, specifically, effective digestive and absorptive functions of the intestinal brush border membrane, and the absence of GI chronic conditions such as enzyme deficiencies, intestinal bower disease, coeliac disease, colorectal cancer and others. In addition, well-balanced intestinal microbiota was associated with an effective immune system function that is required to maintain the host homeostasis [1]. The intestinal microbiota consists of more than a trillion microorganisms that establish a symbiotic relationship with their host. These microorganisms prevent the colonization of potentially pathogenic microorganisms and regulate the mucosal immune system, and thus assist to maintain an intact intestinal barrier [2]. Thus, the physical barrier of epithelial cells and mucus layer provide the first line of defense by mechanisms such as microbial recognition, antibodies secretion, antimicrobial peptides and mucus production [1]. Therefore, impairments of the physical barrier may enhance the risk of infections, inflammatory intestinal diseases and other diseases occurring outside the intestine such as immune-related and metabolic disorders [1,3]. In this context, certain food and plant origin bioactive compounds were investigated to reduce the risk of the mentioned diseases by acting beneficially on the intestinal health.

Anthocyanins are bioactive water-soluble plant pigments that are responsible for bright colors, such as purple, red and blue, which are presented mainly as glycosides, with the basic structure consisting of an anthocyanidin core attached to sugars and organic acids [4]. The positive effects of anthocyanins and anthocyanin-rich foods are widely described in the literature. These effects are mainly associated with reduced risk of diseases associated with oxidative stress, such as cardiovascular disease [5] and inflammatory diseases such as diabetes mellitus [6], obesity [7] and insulin resistance [8]. In addition, the health-promoting effects attributed to anthocyanins were shown to be associated with the gut microbiota modulation [9]. 

Dietary anthocyanins undergo a specific metabolism with the absorption rate depending on their structure. Briefly, anthocyanins cross the gastric mucosa in their intact form. Thereafter, in the small intestine, mainly in the jejunum, they are absorbed by hydrolytic enzymes as phenolic aglycone. The unabsorbed anthocyanins reach the colon, and are metabolized by the colon microbiota, especially by genera and species that are equipped with enzymes such as β-glucosidase, which are necessary to catalyze the reaction. Intestinal bacteria such as *Bifidobacterium* spp. and *Lactobacillus* spp. possess these enzymes; thus, the anthocyanin metabolism by microbiota and/or their metabolites can modulate the growth of these specific bacteria [9,10,11]. In this sense, the modulation of gut microbiota by anthocyanin increases the short chain fatty acids (SCFA) producing bacteria, which acidifies the intestinal pH and inhibits the pathogenic bacteria proliferation, and SCFA as butyrate act as a fuel to provide energy for epithelial cells, thus improving the intestinal barrier to avoid the translocation of pathogens and antigens [12]. Thereby, it is suggested that the potential beneficial functions of anthocyanins could be indirectly attributed to the gut microbiota modulation and consequent production of metabolites due to bacterial fermentation activities, which improve several parameters related to the intestinal health [9,13]. 

Despite the several positive effects of anthocyanins, there is no consensus in the literature regarding their mechanisms of action on intestinal health in experimental studies. Recently, a systematic review (*n =* 6 studies: 3 in vitro, 2 animals and 1 human trial) verified the effects of anthocyanin supplementation on the gut microbiota composition, showing the proliferation of healthy anaerobic bacterial population and inhibition of pathogenic species [14]. However, a healthy gut is maintained by a set of parameters related to the metabolites of microbial bacteria, intestinal cells’ integrity and the physical barrier [1]. Therefore, the objective of the current systematic review was to investigate the effects of anthocyanins on several parameters of intestinal health in experimental studies, in order to understand the mechanism in which these parameters act in association. It is hypothesized that the supplementation of anthocyanin promotes beneficial changes to the gut microbiota with increased production of metabolites associated with intestinal barrier improvement, which contributes to a healthy gut.

## 2. Materials and Methods

### 2.1. Eligibility Criteria 

The eligibility criteria were based on the PICOS (population, intervention, comparison, outcomes and study design) model strategy. Duplicate studies were excluded, and the search and screening for titles and abstracts were carried out independently by the authors according to the inclusion and exclusion criteria (Table 1).

### 2.2. Information Source

Two researchers independently searched for original articles. PubMed, Cochrane and Scopus were used to search studies performed with animal models that evaluated the effects of anthocyanin on the intestinal health. No time restriction was used. The descriptors were identified based on Medical Subject Headings (MeSH). 

### 2.3. Search Strategy 

The following English search terms were used: (Anthocyanin OR Anthocyanidin OR Anthocyanidins OR Cyanidin OR Delphinidin OR Malvidin OR Peonidin OR Pelargonidin OR Petunidin) AND (intestinal OR gut). Only articles published in English were considered in this review. The last search was performed on 2 June 2020. The first selection of the studies was based on the title and abstract. We excluded review articles, clinical studies, theses, dissertations, book chapters, in vitro experiments and studies published in other languages than English. Further, we excluded studies in which the intake of anthocyanin was associated with other foods, or if the anthocyanin was not measured. Studies were eligible for inclusion if they fulfilled the following criteria: (a) studies conducted with animals; (b) the intervention was the intake of foods’ sources of anthocyanin or supplementation with an extract of anthocyanin; (c) the comparator was the negative control (without the intervention); (d) the outcomes searched were changes related to the intestinal health, mainly: changes in the gut microbiota composition, intraluminal pH, short chain fatty acids, histological parameters of small and large intestine, gene expression of tight junction’s proteins, gene expression of intestinal brush border membrane, integrity of intestinal barrier and intestinal permeability. 

### 2.4. Selection, Data Collection Process and Data Items

After reading and reviewing the selected research articles in full, the data were compared to ensure integrity and reliability. Divergent decisions were resolved by consensus. The eligible outcomes evaluated were broadly categorized as follows:

- Gut microbiota: short chain fatty acids (caecal, fecal or in the serum); intraluminal pH (ileal, caecal or feces); microbial quantification; secretory immunoglobulin A (sIgA);
- Epithelial physical barrier: tight junction proteins; proteins of intestinal brush border membrane; intestinal permeability; plasm endotoxin;
- Intestinal morphology: number of goblet cells; length, height and depth of villi and crypts; mucin secretion; antimicrobial peptides.

Any measure and methodology of these outcomes was eligible for inclusion.

Further, for each experimental study included, we reported relevant information related to the authors, publication year, country of publication and experimental model features such as animal model, age, sex, initial weight, number of groups and animal per group. To access the research methods, we extracted specific information related to the experimental groups such as type of food intervention, type of diet and control group. For the control test of food intake, we extracted information related to the method of administration that was used in the intervention, the duration of the intervention, the dosage of anthocyanin and main results (control x intervention). 

For this review, data from the eligible studies are expressed in tables and figures. We provided a narrative synthesis of the results according to the main characteristics and results.

### 2.5. Study Risk-of-Bias Assessment 

The methodological quality of the included studies was assessed, and the risk of bias was verified using the Systematic Review Centre for Laboratory Animal Experimentation Risk of Bias (SYRCLE RoB) tool [15], which is responsible for identifying study quality and measuring the bias in research involving animal studies [16]. The SYRCLE RoB toll considers ten entries that are related to six types of bias: selection bias, performance bias, detection bias, attrition bias, reporting bias and other. For each included study, the six bias types were classified as “high” (+), “low” (−) or “unclear” (?).

## 3. Results

### 3.1. Study Selection 

**Figure 1:**
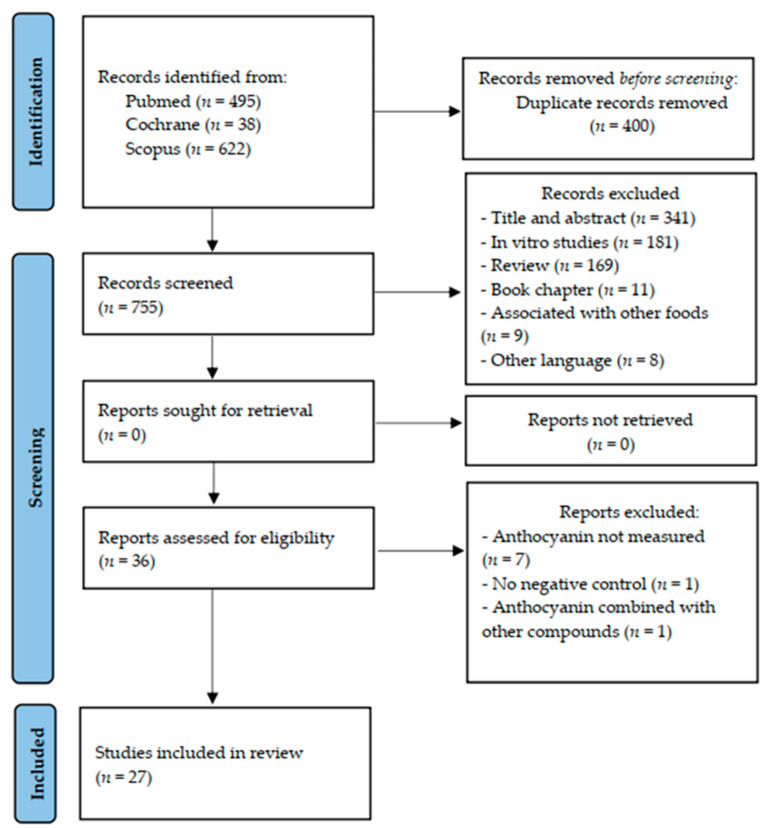
Flowchart of the search for articles included in the systematic review, according to PRISMA (2020) recommendation.

The flow diagram of the literature search and selection process was built in accordance with the Preferred Reporting Items for Systematic Reviews and Meta-Analyses (PRISMA) guideline (Figure 1). After the search in the selected databases, we identified 1155 articles (x = 495 Pubmed; x = 38 Cochrane and x = 622 Scopus). From these, 1117 were excluded for the following reasons: duplicate studies (*n =* 400), title and abstract not suited to the topic (*n =* 341), in vitro studies (*n =* 181), review articles (*n =* 169), book chapters (*n =* 11), supplementation of anthocyanin associated with other foods (*n =* 9), and other languages than English used (*n =* 8). After, 36 articles were read fully. From these, we excluded nine articles: total of anthocyanin not measured (*n =* 7); there was no negative control without intervention (*n =* 1); and anthocyanins were combined with other compounds (*n =* 1). Therefore, 27 studies were included in this systematic review.

### 3.2. Study Characteristics 

The included studies (*n =* 27) were performed in ten different countries. Most of them were conducted in China (*n =* 9) [17,18,19,20,21,22,23,24,25] or the United States of America (U.S.A.) (*n =* 5) [26,27,28,29,30]. Regarding the animal model used in the studies, 15 were performed with mice [17,20,21,22,24,25,26,27,28,29,30,31,32,33,34], 11 with rats [18,19,23,35,36,37,38,39,40,41,42] and only 1 with rabbits [43]. Most studies used male animals (*n =* 24) [17,18,20,21,22,23,24,25,26,27,28,29,31,33,34,35,36,37,38,39,40,41,42,43], but two used female animals [19,30], and only one study used both male and female animals [32]. Interesting, six studies did not describe animals’ initial weight [19,21,28,30,32,38]. The age of the animals ranged from 3 to 72 weeks, although five studies did not mention this information [17,27,35,36,39]. The studies’ main characteristics were chronologically organized by the publication year, starting with the first published (Table 2).

The anthocyanin intervention varied by the source that was used. In 20 studies, diverse fruits were offered in the form of extract (*n =* 12) [17,19,25,30,32,33,34,35,36,40,41,43], or as a powder (*n =* 8) [22,26,28,31,37,38,39,42]. The fruits used as an anthocyanin source were chokeberry, Kamchatka berry, apple, blackcurrant, bilberry, blueberry, black raspberry, red cabbage, grape, berry juçara, jabuticaba, purple carrot, black gojy berry and black rice. In four studies, the intervention was with purified anthocyanin [20,23,24,27], and in three studies with monomeric anthocyanidins as cyanidin-3-O-glucoside [18], malvindin-3-glucoside [29] and pelargonidin-3-O-glucoside [21]. 

The form of anthocyanin administration was oral in all included studies, with the form of administration by addition in the diet (*n =* 20) [18,22,24,25,26,27,28,29,30,31,32,33,35,36,37,38,39,40,42,43], or via gavage (*n =* 5) [17,19,21,23,34] or drinking water (*n =* 2) [20,41]. The duration of interventions ranged widely from 1 to 20 weeks. Regarding the anthocyanin dosage, the doses observed varied from 12.9 mg/100 g diet [42] to 1280 mg/100 g diet [40], and 1.68 mg/kg body weight (BW) [34] to 200 mg/kg BW [20,24]. In addition, only one study offered a aqueous extract in the dose of 75 mg/L [41] (Table 3).

### 3.3. Main Findings

The reviewed experimental studies demonstrated that the anthocyanin supplementation provided beneficial effects to intestinal health, and specific improvement in the intestinal microbiota population, short chain fatty acids production, goblet cell number, tight junction protein and villi improvement (Table 3). 

Positive findings included the effects on the intestinal microbiome composition and function. In this context, the majority of the studies observed an increased abundance of *Bacteroidetes* [22,23,26,28,30,33]; two studies observed a reduction [18,39]; and one showed no changes on the abundance of *Bacteroidetes* [20]. On the other hand, the abundance of *Firmicutes* was reduced in five studies [21,22,23,28,39], and in two studies no changes were observed [20,30]. Further, a reduction in the *Firmicutes*/*Bacteroidetes* ratio (total of studies that evaluated = 7) was observed in four studies [19,25,27,30];in two other studies, no changes were observed [20,34], and only one study observed an increased ratio [29]. Further, an increase in *Biffidobacterium* spp. and *Lactobacillus* spp. populations were observed in some of the studies [19,23,25,38,40]. 

Out of all the studies included, 12 evaluated the production of short chain fatty acids (SCFA) by bacterial populations. This analysis indicated an increased total SCFA production in most of the studies (*n =* 7) [19,20,21,22,24,25,42]. Further, four studies reported on a reduction of the intestinal or cecal pH [22,35,42,43]. Moreover, regarding proteins that are related to intestinal permeability and function, most of the studies observed an increase in the gene expression of zonula occludents 1 (ZO-1), occludin, claudin-1 and Mucin (Muc) 2 [20,21,22,24,25,27,39], and three studies did not observe these effects [33,34,38]. The intestinal morphology was evaluated in some of the studies. From seven studies, which evaluated the number of goblet cells, five studies observed an increased number [17,20,24,25,39], and in the other two studies no changes were observed [34,40]. In addition, increased villi length or height was reported in many of the studies [17,18,22,23,25,39]. Four studies [19,21,22,39] observed a reduction in the serum lipopolysaccharides (LPS), and one study [27] showed a reduction in plasma endotoxin and intestinal permeability.

### 3.4. Risk of Bias 

**Figure 2:**
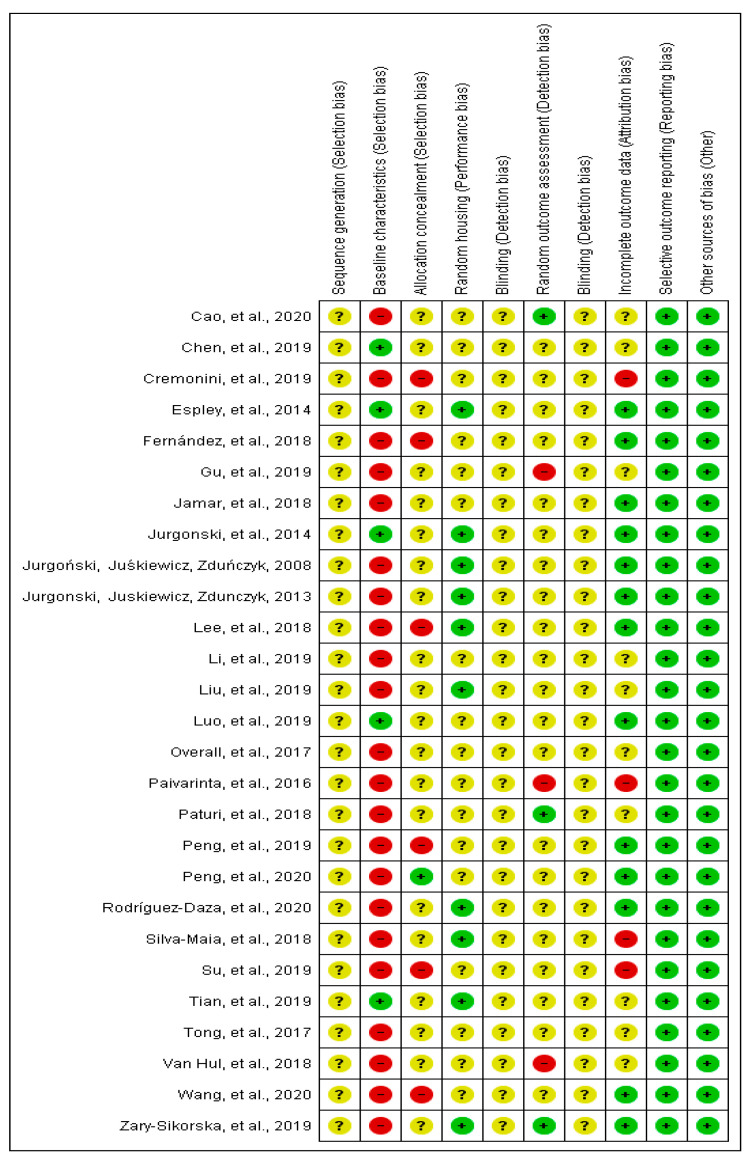
Risk of bias of animal studies.

From all the studies that were included in the current systematic review (*n =* 27), the baseline characteristics, including sex, age and initial weight of animals, were complete in five studies [18,22,23,31,43]. In most of the studies, the allocation of animals was not described in detail, since there was no information about the randomization process. Six studies did not mention if the animal allocation to treatment groups was performed randomly [20,21,25,27,37,39]. Furthermore, none of the studies reported about blinding the investigators involved in the research. Four studies did not include all animals in the analysis, and the exclusion criteria were not reported [21,27,32,41] (Figure 2).

## 4. Discussion 

In this systematic review, we evaluated the effects of anthocyanins or their extract on intestinal health parameters, in vivo. Therefore, this systematic review verified that food sources of anthocyanin or its extract are able to improve intestinal parameters changed by pathologic conditions or dietary patterns. 

The animal models of the studies included were high fructose diet, high fat diet, intestinal mucositis, colorectal cancer, damage to the intestinal mucosa, diabetes and old animals. It is highlighted that all of these models promote intestinal changes, such as increased intestinal permeability, inflammation, altered morphology and changes in the intestinal microbiota composition such as dysbiosis. Despite different animal models being used in the studies, all of them except one [43] used rodents as the animal. Similarly as in humans, *Bacteroidetes* and *Firmicutes* are the two main phyla in rodents’ gastrointestinal tract. In this sense, microbiota composition in rodents is usually analyzed in interventions that study the casual role of gut microbiota in diet, health and disease interaction [44]. Since all animal models used in the studies of this review promote modification of the gut microbiota at some level, the choice of the best model depends on the main goal of the study [44]. Therefore, bioactive components with functional properties were investigated to verify their potential beneficial effects on intestinal health [7]. Anthocyanins are soluble components, from the class of flavonoids, with functional dietary properties that were previously associated with oxidative stress inhibition, antioxidant activity and intestinal microbiota modulation [45]. Further, the supplementation of anthocyanin cyanindin-3-O-glucoside for eight weeks in Wistar rats was able to restrain the gut microbial dysbiosis that was induced chemically, by suppressing the decrease of *Rothia* and *Romboutsia* and the increase of *Clostridium* verified in the disrupted gut microbiota [18]. 

In this review, the anthocyanin dietary intake induced increased abundance of *Bacteroidetes* and a reduction of *Firmicutes*. A reduction of the *Firmicutes*/*Bacteroidetes* ratio in the caecal content was observed in experimental in vivo models of high fat diet [25,27]. In recent years, research with animal models in samples of caecal content [23] and humans in fecal samples [46] demonstrated that obese organisms have a high abundance of *Firmicutes* and a low abundance of *Bacteroidetes*. These changes in the microbial composition result in an increased absorption of calories, reduced secretion of anorexigenic hormones and intestinal barrier damage [46]. *Bacteroidetes* and *Firmicutes* are the two main phylum that inhabit the large intestine, corresponding to 90% of total bacteria [47]. The *Firmicutes* phylum and gram-positive bacteria carry more enzymes that are required for carbohydrates metabolism, which contribute to their transport and energy absorption [48], besides higher fat deposition in adipocytes [49]. 

Following anthocyanin consumption, most are not absorbed in the upper gastrointestinal tract, and therefore reach the colon intact [11]. At the colonic level, anthocyanins are metabolized by the local microbiota, initially via deglycosylation, and is followed by a secondary degradation into phenolic acids, mainly protocatechuic, vanillic, syringic, gallic and *p*-coumaric [45]. The main bacterial populations that are able to metabolize anthocyanin are the *Bifidobacterium* spp. and *Lactobacillus* spp., which have probiotic effects, including the production of antimicrobial substances, competition with pathogens for adhesion to the epithelium and nutrients, immunomodulation and inhibition of bacterial toxin production [50]. In addition, these bacteria have enzymes such as β-glycosidase that are needed to catalyze reactions that release the glycose from the aglycon and provide the energy needed for bacterial populations to prosper [12]. In several studies included in this review, an increase of *Bifidobacterium* and *Lactobacillus* was documented [19,23,25,38,40]. In this context, 20 day intake of dealcoholized red wine in healthy adults increased the fecal concentration of *Bifidobacterium, Enterococus* and *Eggerthella lenta*. In addition, in this study, the produced metabolites associated with the increase of *Bifidobacterium* were those derived from anthocyanin degradation (4-hydroxybenzoic, syringic, *p*-coumaric, homovanillic ácidos) [51]. *Bifidobacterium* is related to pathogen inhibition by organic acids production, antimicrobial peptides and immune stimulation [52]. Besides this, among the acids produced by the microbial metabolism of anthocyanin, protocatechuic acid presents inhibitory effect on pathogenic bacteria growth [53], and gallic acid is effective in reversing changes in the microbiota caused by induced colitis in animals, by reducing *Firmicutes* and *Proteobacteria* and increasing *Bacteroidetes* [54]. Therefore, the beneficial effects that are associated with anthocyanin consumption may be achieved by its metabolites post-degradation. 

Some of the physiological properties of the gut microbiota are attributed to fermentation of non-digestible carbohydrates by anaerobic bacteria, producing short chain fatty acids (SCFA) [47]. In this review, the dietary supplementation of anthocyanin was able to increase the production of total SCFA (acetate, propionate and butyrate) in the majority of the studies. SCFA act as a fuel to intestinal cells by stimulating the cellular proliferation. The production of SCFA also reduces the intraluminal pH, which limits the growth of pathogenic bacteria due to the acidification [55,56]. Therefore, SCFA assist to maintain the intestinal epithelium integrity, and protect the host from potential immune and inflammatory diseases [56]. In a mice study, where diet included anthocyanin extract, SCFA production increased due to elevated microbial activity, specifically of *Bifidobacterium* and *Lactobacillus* [25]. Further, the increased abundance of *Roseburia* is associated with a higher production of SCFA in the intestine, and the abundance of *Akkermansia* was associated with propionic production [23]. SCFA also have an immunomodulatory effect by promoting the development of mucosal regulatory T cells (Tregs) through the interaction with the G protein-coupled receptor (GRP43) and the inhibition of histone deacetylases (HDACs) [57,58]. Furthermore, SCFA exert positive effects to the turnover and differentiation of colonic epithelial cells, and to stimulate the mucus production that prevents the pathogenic bacteria adherence [56]. It is suggested that these acids activate the mammalian target of rapamycin (mTOR) complex and the STAT3 (signal transducers and activator of transcription 3) in the intestinal epithelial cells, which promote the expression of antimicrobial peptides as β-defensin and RegIIIγ [59]. From the included studies in this systematic review, two observed an increase of Lysosome-1 (Lyz1) peptide, which acts to maintain the microbiota homeostasis and to eliminate commensal microorganisms [21,33]. Therefore, by these mechanisms, SCFA act to maintain the epithelial barrier integrity [60,61].

The intestinal epithelial barrier consists of a mucus layer and cells attached by a protein complex, including tight junctions, adherents junctions and desmosome [62]. The tight junctions complex is composed by proteins as claudins, occludin, junctional adhesion molecule (JAM-1) and zonula occludents (ZO-1), and the rupture in some of these proteins increases the paracellular permeability with permeation of pro-inflammatory molecules, immune activation and inflammation [63]. In this review, the majority of the included studies verified an increase in the gene expression of ZO-1, occludin and claudin 1. The anthocyanin has an anti-inflammatory effect through inhibition of the factor nuclear kappa B (NF-ĸB), and via regulation of I-Kappa-B-alpha (IĸBα) phosphorylation that decreases the gene expression of pro-inflammatory cytokines, such as tumor necrosis factor alpha (TNF-α), interferon gamma (IFNγ) and interleukins [64,65]. These cytokines are related to intestinal barrier damage by harming the tight junction protein expression [66]. The TNF-α promotes changes in the tight junctions via its receptor tumor necrosis factor receptor 1 (TNFR-1), so that anti-TNF strategies promoted tight junctions’ rearrangement with an improvement of occludin and ZO-1 [67]. Therefore, the anthocyanidin cyaniding-3-glucoside (C3G) was able to inhibit the IĸBα phosphorylation and the nuclear translocation of NF-ĸB (p65), and these effects were associated with the nuclear transcription factor Nrf2 (erythroid-2-related factor 2) that induces the expression of antioxidant enzymes [68]. Therefore, the anthocyanin can act directly or indirectly to improve tight junction’s integrity. 

Moreover, the intestinal epithelial cells are covered by a mucus layer produced and secreted by the goblet cells. The mucus is composed of glycoproteins of mucins, mainly Muc2, forming a viscous layer that protects against pathogen invasion by preventing the sites of binding for bacteria [69,70]. The discontinuous mucus layer in the cecum of rats are considered hotspots for *Salmonella*; thus, the absence of the mucus layer can lead to infections’ development [71]. In addition, the increase in goblet cell number, mainly those classified as acids, promotes barrier function improvement by increasing the mucin secretion that prevents pathogen invasion and possible intestinal inflammation due to acidic mucin resistance to degradation by bacterial glycosidase and has higher viscosity [72]. SCFA also contribute to the intestinal cell proliferation since they stimulate the proliferation and differentiation of enterocytes [73]. Thus, there is evidence that the inflammasome nucleotide-oligomerization domain-like receptor 6 (NLRP6), expressed mainly on enterocytes, controls the mucin secretion and the mucosa renewal by the goblet cells [74]. A previous study with rats suggested that SCFA can activate the colonic NLRP6, thus protecting the intestinal barrier [75]. Considering this evidence, it is verified that the mulberry (50 mg anthocyanin/kg diet) supplementation in animals with induced colitis resulted in an increase in goblet cells and NLRP6 expression, therefore suggesting a link between mucin secretion and antimicrobial peptide production [76]. 

In order to optimize the digestion and absorption of nutrients, the gut (duodenum) morphology is unique and organized in villi [77]. It is observed that anthocyanins are related to an improvement of the absorptive function by increasing the villus length, villus length/crypt depth ratio and the total mucosa thickness [78]. These intestinal morphology changes result in a better intestinal digestion and absorption, since they assure a higher absorption surface, brush border enzyme expression and nutrient transport system [79]. 

Disruption of the intestinal barrier integrity can be occasioned by tight junction disruption and mucus layer depletion, thus allowing the paracellular translocation of bacteria and their components, such as lipopolysaccharides (LPS) [63]. Of the included studies in this systematic review, a few evaluated the endotoxemia; however, of these, the anthocyanin supplementation was able to reduce the intestinal permeability, endotoxemia and the levels of serum LPS [19,21,22,27,39]. LPS are cellular wall components of gram-negative bacteria that contain a pathogen-associated molecular pattern, Lipid A, able to interact with Toll-like Receptor 4 (TLR-4) via the Myeloid differentiation primary response 88 (MyD88) protein [80]. This interaction results in the activation of the pathway downstream and Nf-ĸB translocation, thus increasing the gene transcription of cytokines such as TNF-α, IL-1β e and IL-6 [80,81]. The analysis of monocyte from obese individuals supplemented with berry juçara (5 g/day; 131.2 mg total anthocyanins) for six weeks observed the reduction of mRNA (messenger ribonucleic acid), TLR4 and the protein expression of MyD88 [82]. Therefore, it is known that the inflammation mediated by LPS can exert local and systemic effects and be related to gastrointestinal diseases, such as Crohn disease [83], inflammatory bower disease [84] and metabolic disorders as diabetes *mellitus* type 2 [85] and obesity [86]. 

Animal experiments assist to design clinical studies in terms of doses, duration, type of intervention and other topics [15]. In this context, the positive changes observed at the gut microbiota following anthocyanin supplementation and consumption in the animal studies included in this review corroborate with results verified in several clinical studies. Cranberry consumption (30 g, with 83.7 mg anthocyanin) for five days by healthy adults resulted in increased abundance of *Bacteroidetes* and decreased abundance of *Firmicutes* [87]. Further, the intake of dealcoholized red wine for 20 d (9.72 mg anthocyanin) increased the fecal concentration of *Bifidobacterium* and *Enterococus* [51]. Thus, we highlighted the complexity of animal models of gut microbiota, which are able to tolerate the presence and effects of dietary components such as anthocyanins in a similar manner as described in human studies. Further, animal model studies related to anthocyanin supplementation are effective in demonstrating the safety and efficacy of their consumption. Hence, these aspects are relevant and important for the translation of results and adaptation to clinical studies, and in order to establish dietary guidelines for humans.

Finally, systematic reviews guarantee the gathering of evidence related to a specific topic; therefore, they obtain conclusions with greater scientific rigor. The evidence verified in this review, performed with 27 studies, demonstrates that the anthocyanin dietary intake is beneficial and improves specific parameters such as the gut microbiota composition, short chain fatty acids production and the intestinal physical barrier, such as the increase of tight junction protein and goblet cell number, and the reduction of intestinal permeability, that together promote intestinal health. 

### Dosage and Reporting Quality 

This systematic review showed high heterogeneity among the studies, with several experimental models used, distinct methodologies in the intestinal parameters analyzed and high variation related to dose and time of anthocyanin supplementation. Probably, these variations were observed because of the large number of studies included. The anthocyanin supplementation dose ranged from 12.9 mg/100 g diet as dried purple carrot [42] to 1280 mg/100 g diet as extract [40]. The supplementation as an extract allows for the delivery of a higher dose of anthocyanins, since it concentrates the components, while the anthocyanin intake in its food source provides a lower dose, based on average daily intake of the animal. Hence, the safety of anthocyanin intake were tested and approved in animals, indicating no toxicity or any adverse effects to animal health, even at a high dosage [88]. In addition, studies showed that the anthocyanin supplementation exposure period varied from 1 week [17,38] to 20 weeks [37]. Analyzing the outcomes individually, the time of supplementation from 1 to 12 weeks promoted the increase of *Bifidobacterium* and *Lactobacillus*, the production of SCFA and an improvement of goblet cells and villi length or height; further, the increase of proteins related to intestinal permeability was verified from the time of 1 to 14 weeks of intervention. Thus, for the design of studies for new researches evaluating the effects of anthocyanin on intestinal health, this range of time should be considered in accordance with the goal of the research. Beneficial effects of anthocyanin supplementation were observed even in the lowest dosage [42] and the shortest exposure period [17,38]. Furthermore, most of the reviewed studies observed that anthocyanin was quantified as total anthocyanin; however, few studies showed its profile, as specific monomers of anthocyanidin such as cyanidin galactoside [31], cyanidin-3-O-glucoside [18], malvindin 3-glucoside [29] and pelargonidin-3-O-glucoside [21]. 

This systematic review evaluated the effects of dietary anthocyanin in the context of its potential intestinal health-promoting effects, such as beneficially changing the intestinal microbiota, increasing the short chain fatty acid production, reducing the intestinal permeability and improving parameters related to the intestinal physical barrier such as tight junctions protein and goblet cell number. The studies’ selection was based on methodologies that are recommended and approved for systematic review, thus allowing reliable conclusions. The risk of bias was evaluated according to the SYRCLE RoB tool [15], which establishes consistency and avoids discrepancies to evaluate the risk of bias from animal studies. Twenty-one (*n =* 21) studies did not show the animals’ completed baseline characteristics, and none of the studies showed information about whether researchers were blinded from knowledge of the intervention groups and/or the outcome assessor. In this sense, the absence of some baseline characteristics probably had no influence on the main conclusion of this review, since those characteristics were not comparable among studies. On the other hand, the risk of bias related to blind researchers and outcome assessors could have influenced the results of outcomes in each study. However, the conclusion of this review was performed with a large number of studies in association; thus, these biases may not represent a major impact on the main conclusions, considering the methodologic rigor that this review followed. Further, the risk of bias in analysis may represent a lack of information regarding the experimental design of animal studies, showing that progress is needed in this field. Therefore, we suggest that research performed with animals follows the SYRCLE protocol to avoid a lack of information in the studies.

## 5. Conclusions

**Figure 3:**
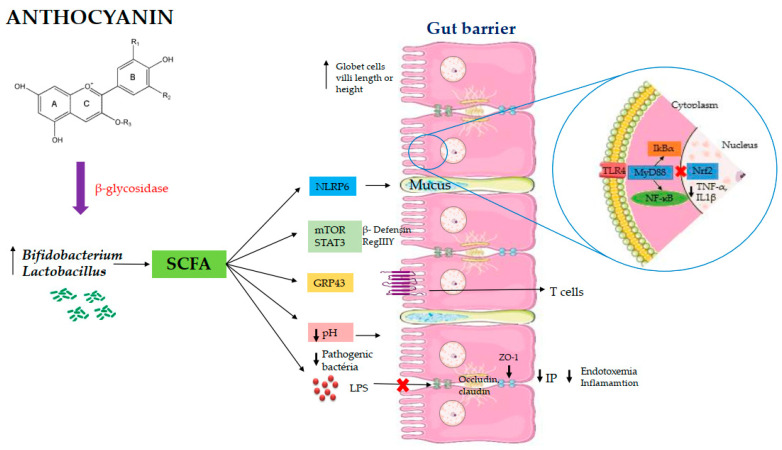
Proposed mechanisms of action of anthocianins on intestinal health. Abbreviations: ZO-1: zonula occludentes–1; SCFA: short chain fatty acids; LPS: lipopolysaccharides; TLR-4: Toll like receptor 4; IP: intestinal permeability; mTOR: mammaliam target of rapamycin; STAT3: signal transducers and activator of transcription 3; NF-ĸB: factor nuclear kappa B; MyD88: Myeloid differentiation primary response 88; IkBα: I-Kappa-B-alpha; NLRP6: inflammasome nucleotide-oligomerization domain-like receptor 6; GRP43: G protein-coupled receptor; TNF-α: tumor necrosis factor alpha IL1β:interleukin 1 beta; Nrf2: erythroid-2-related factor.

The scientific evidence from the reviewed in vivo studies demonstrates that the supplementation of anthocyanin is effective to modulate the intestinal microbiota through the increase of *Lactobacillus* spp. and *Bifidobacterium* spp., and to increase the production of short chain fatty acids. In addition, the reviewed studies observed an improvement in the intestinal barrier by the increased expression of tight junction protein associated with an improvement of cells’ morphology and mucus production, which reduces the potential risk of inflammation (Figure 3). We highlighted that these intestinal changes in association may be the mechanisms by which upon anthocyanin supplementation that ranged from 1 to 14 weeks with the dosage that ranged from a 12.9 to 1280 mg/100 g diet exert beneficial effects on intestinal health. We consider that it is not adequate to establish a specific dose and a specific time to achieve all of these effects in association, since different animal models, methodologies and large ranges of time and dose of anthocyanin supplementation were observed. However, considering the methodological rigor that this review followed, the dose and time intervention ranges observed could be used as guidelines for future researchers. Despite the limitation of extrapolating animal results to human, with knowledge of all of the benefits observed, we consider that the daily intake of foods’ source of anthocyanin should be stimulated, with the population acting as a strategy to prevent health problems. 

### Registration and Protocol

This systematic review was realized according to the protocol: *Preferred Reporting Items for Systematic Reviews and Meta-Analyses: The PRISMA Statement 2020* [89]. The review is registered in the PROSPERO under the number CRD42020204835 (Centre for Reviews and Dissemination, University of York). A systematic review was carried out to answer the question: “Does food source or extract of anthocyanin promote changes on intestinal parameters?”

## Figures and Tables

**Table 1:** PICOS criteria for inclusion and exclusion of studies.

Parameter	Inclusion Criteria	Exclusion Criteria
Population	In vivo animal studies	Clinical studies and in vitro studies
Intervention	Intake of foods’ source of anthocyanin or supplementation with extract of anthocyanin	Anthocyanin associated with other foods or not measured
Comparator	Negative control (without the intervention)	No control group
Outcomes	Changes in the gut microbiota composition, intraluminal pH, short chain fatty acids, histological parameters of small and large intestine, gene expression of tight junction’s proteins, gene expression of intestinal brush border membrane, integrity of intestinal barrier and intestinal permeability	
Study design	Experimental placebo-controlled studies	Review articles, clinical studies, theses, dissertations, book chapters, in vitro experiments and studies published in other languages than English.

**Table 2:** Characteristics of animal studies on the effects of anthocyanin on intestinal health.

Author, Year	Country	Animal Model/Age	Sex	Initial Weight (g)	Nº of Groups	Nº of Animals/Groups
Jurgonski, Juskiewicz, Zdunczyk, 2008 [35]	Poland	Wistar rats/NS	Male	161 ± 8	3	8
Jurgonski, Juskiewicz, Zdunczyk, 2013 [36]	Poland	Wistar rats/NS	Male	548 ± 36	3	8
Espley, et al., 2014 [31]	New Zealand	Swiss mice/6–7 wk	Male	30	3	10
Jurgonski, et al., 2014 [43]	Poland	White rabbits/34 days	Male	631 ± 26	4	5
Paivarinta, et al., 2016 [32]	Finland	C57BL/6J Apc^mim^ mice/5 wk	Male and female	NS	3	5–6 male and 4–6 female
Overall, et al., 2017 [26]	U.S.A.	C57BL/6J mice/6 wk	Male	20–30	8	12 or 8
Tong, et al., 2017 [17]	China	Kunming mice/NS	Male	22 ± 5	5	10
Fernández, et al., 2018 [37]	Spain	Fischer 344 rats/5wk	Male	200–270	3	10
Jamar, et al., 2018 [38]	Brazil	Wistar rats/90 days	Male	NS	3	7
Lee, et al., 2018 [39]	Georgia	Wistar rats/NS	Male	200–220	3	8
Paturi, et al., 2018 [40]	New Zealand	Sprague-Dawley rats/3 wk	Male	256–265	8	16
Silva-Maia, et al., 2018 [41]	Brazil	Wistar rats/3 wks	Male	0–100	3	5 or 8
Van Hul, et al., 2018 [33]	France	C57BL/6J mice/9 wk	Male	25–30	4	14
Chen, et al., 2019 [18]	China	Wistar rats/13 wks	Male	403 ± 4	5	8
Cremonini, et al., 2019 [27]	U.S.A.	C57BL/6J mice/NS	Male	20–25	4	10
Gu, et al., 2019 [28]	U.S.A.	C57BL/6J mice/4 wk	Male	NS	2	12 or 14
Li, et al., 2019 [19]	China	SD rats/4 and 12 months	Female	NS	6	10
Liu, et al., 2019 [29]	U.S.A.	C57BL/6J mice/4–5 wk	Male	18–21	3	9 or 10
Luo, et al., 2019 [23]	China	Sprague-Dawley rats/4 wk	Male	100–120	6	8
Peng, et al., 2019 [20]	China	C57BL/6J mice/5 wk	Male	21–24	4	10
Su, et al., 2019 [21]	China	*db/db* mice C57BL/6J/6 wk	Male	NS	2	12
Tian, et al., 2019 [22]	China	C57BL/6J mice/4 wk	Male	15–18	6	11
Zary-Sikorska, et al., 2019 [42]	Poland	Wistar rats/13 wk	Male	146 ± 1.051	5	8
Cao, et al., 2020 [30]	U.S.A.	C57BL/6J mice/3–18 months	Female	NS	4	3
Peng, et al., 2020 [24]	China	C57BL/6J mice/5 wk	Male	20–24	2	10
Rodríguez-Daza, et al., 2020 [34]	Canada	C57BL/6J mice/6 wk	Male	20–25	6	12
Wang, et al., 2020 [25]	China	C57BL/6J mice/6 wk	Male	19–20	5	12

NS: not specified; wk: weeks; U.S.A.: United States of America; Apc.: adenomatous polyposis coli.

**Table 3:** Main findings in animal studies on the effects of anthocyanin on intestinal health.

Reference	Design (Intervention)	Control	Administration/Duration of Intervention (Weeks)	Method of Gut Microbiota Evaluation/Type of Sample	Anthocyanin Dosage (Total Anthocyanin)	Main Results (Intervention × Control)
Jurgonski, Juskiewicz, Zdunczyk, 2008 [35]	Chokeberry fruit extract (0.2%) + High fructose diet and streptozotocin	High fructose diet and streptozotocin	Oral (diet)/4	NA	80.9 mg/100 g diet	↓ ileal pH; Mucosal disaccharidase activity: ↓ sucrase and maltase and ↑ lactase; 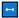 Total SCFA; 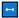 cecum pH; 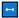 α- and β- glucosidase, α- and β- galactosidase and β-glucuronidase on cecum.
Jurgonski, Juskiewicz, Zdunczyk, 2013 [36]	Kamchatka berry extract (2g/kg diet) + Diet with fructose replaced the corn starch	Diet with fructose replaced the corn starch	Oral (diet)/4	NA	65.4 mg/100 g diet	Mucosal disaccharidase activity: 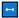 sucrase, maltase and lactase; 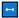 cecum pH; ↑ α- and β- glucosidase on cecum; 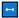 α- and β- galactosidase on cecum; 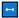 ileal pH.
Espley, et al., 2014 [31]	Freeze-dried apple (20%) + Normal diet	Normal diet	Oral (diet)/3	qPCR Colonic content	397 µg/g diet *	↑ Total bacteria; ↓ *Lactobacillus* spp.; 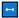 *Bifidobacterium* spp.; 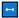 *Bacteroides-Prevotella-Porphyromonas* group.
Jurgonski, et al., 2014 [43]	Blackcurrant pomace extract (1.5%) + HFD	HFD	Oral (diet)/4	NA	733.5 mg/100 g diet	↓ Small intestine pH; 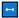 Caecum pH; ↓ β-glucuronidase; 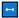 α- and β- glucosidase, α- and β- galactosidase; 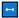 Total SCFA cecal.
Paivarinta, et al., 2016 [32]	Bilberry extract (10%) + HFD	HFD	Oral (diet)/10	PCR-DGGE Cecum content	553.2 mg/100 g diet	↑ Bacterial diversity in cecal contents.
Overall, et al., 2017 [26]	Blueberry powder (400 µg/g total anth.) + HFD	HFD	Oral (diet)/12	qPCR Fecal sample	40 mg/100 g diet	↑ Abundance of *Bacteroidete* and *Actinobacteria*.
Tong, et al., 2017 [17]	Anthocyanin from red cabbage extract (100mg/kg BW) + CPT-11 (to induce intestinal mucositis)	CPT-11 (to induce intestinal mucositis)	Oral (gavage)/1	NA	100 mg/kg BW	↑ Goblet cell mucus; Preservation of the villi height and conserved epithelial cell surface in the ileum and colon.
Fernández, et al., 2018 [37]	Functional sausage (20g with 0.11% anth.) + AOM treatment (to induce CRC tumor)	AOM treatment + Control sausage (20g)	Oral (diet)/20	NGS Caecal feces	22 mg/20 g sausage	↓ Hyperplastic payer patches in the small intestine mucosa; ↓ level of Desulfovibrionaceae and Enterobacteriaceae and ↑ of Clostridiaceae; ↓ *Bilophila wadsworthia.*
Jamar, et al., 2018 [38]	Juçara powder (0.25%) + HFD	HFD	Oral (diet)/1	qPCR Colon content	1.65 mg/kg/day	↓ mRNA of TLR-4 in the colon; 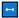 mRNA ZO-1; ↑ DNA levels of *Bifidobacterium* spp.
Lee, et al., 2018 [39]	Blueberry powder (10%) + HFD	HFD	Oral (diet)/8	NGS Caecal content	213.4 mg/100 g diet	↓ *Bacteroidetes* and *Firmicutes* abundance; ↑ Abundance of *Proteobacteria* and *Fusobacteria*; ↑ *Bacilli* and *Lactobacillales;* ↑ mRNA *Muc*2 ileal; ↑ ileal villus length and goblet cell number; ↑ serum acetate; 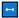 Serum propionate and butyrate; ↓ serum LBS (to assess LPS concentration); 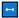 mRNA antimicrobial peptide Defb2.
Paturi, et al., 2018 [40]	Blackcurrant extract (40g/kg) + Control diet	Control diet	Oral (diet)/6	qPCR Caecal content	1280 mg/100g diet	↓ cecal acetic and butyric and ↑ of propionic acid; ↑ *Bacteroides-Provotella-Porphyromonas* group and *Lactobacillus* spp.; ↓ *Bifidobacterium* spp. and *Clostridium perfringens*; 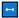 crypt depth and goblet cells in the colon.
Silva-Maia, et al., 2018 [41]	Aqueous extract of berry (*Plinia jaboticaba*) peel (50g/L) + Normal diet	Normal diet	Oral (water)/7	Colonies expressed as CFU Colon content	75 mg/L	↑ *Enterobacteriaceae* and *Bifidobacterium*, and 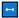 *Lactobacillus*; 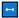 total SCFA.
Van Hul, et al., 2018 [33]	Grape pomace extract (8.2 g/kg diet) + HFD	HFD	Oral (diet)/8	NGS Caecal content	35.59 mg/100 g diet	↑ Abundance of *Bacteroidetes*; ↓ *Desulfovibrionaceae* and *Spreptoccaceae*; ↑ *Prevotellaceae* and *Erysipelotrichaceae*; 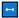 mRNA of ZO-1, intectin, occludin, claudin3, Muc2, Reg3ϒ; ↑ mRNA Lyz1; 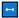 Total SCFA cecal.
Chen, et al., 2019 [18]	Purified cyanidin-3-O-glucoside (1000mg/kg) + 3-MCPD	3-MCPD (to damage the intestinal mucosa)	Oral (diet)/8	NGS Colonic content	1000 mg/kg diet **	↓ *Bacteroidetes* levels and ↑ *Proteobacteria* and *Actinobacteria*; ↑ Villus height, and number of epithelial cells.
Cremonini, et al., 2019 [27]	Anthocyanin rich mix (40mg/kg) + HFD	HFD	Oral (diet)/14	NGS Caecal content	40 mg/kg BW	↓ Intestinal permeability; ↓ Plasm endotoxin; ↓ ratio *Firmicutes*/*Bacteroidetes*; ↑ *Romansia* abundance; ↑ Protein expression of occludin, ZO-1 and claudin-1; ↑ Muc2 secretion.
Gu, et al., 2019 [28]	Black rasberry powder (10%) + Control diet	Control diet	Oral (diet)/6	NGS Luminal content	290 mg/100 g diet	↓ Abundance of Firmicutes and ↑ of Bacteroidetes; ↓ *Clostridium* ↑ *Barnessiella*
Li, et al., 2019 [19]	Bilberry anthocyanin extract (20 mg/kg) + Old rats	Old rats	Oral (gavage)/10	NGS Caecal content	20 mg/kg BW	↓ Abundance of *Verrucomicrobia* and *Euryarchaeota;* ↓ Ratio *Firmicutes/Bacteroidetes*; *↑* Species of *Weissella confuse* and *Aspergillus oryzae;* ↑ *Lactobacillus and Bacteroides*; ↑ Total SCFA in cecal content; ↓ β-glucosidade and α-galactosidade and 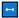 α-glucosidase, α-galactosidade, and β-glucoronidase; ↓ serum LPS.
Liu, et al., 2019 [29]	Malvindin 3-Glucoside (24mg/kg diet) + DSS	DSS	Oral (diet)/50 days	NGS Colon content	24 mg/kg diet ***	↓ Abundance of *R. gnavus* and ↑ *Clostridium* and *Bacteroides ovatus*; ↑ *Firmicutes*/*Bacteroidetes* ratio; ↑ crypt dilation.
Luo, et al., 2019 [23]	Purified anthocyanin from *L. ruthenicum* (200 mg/kg BW) + HFD + vit. D3	HFD + vit. D3 (to induce atheroscherosis)	Oral (gavage)/6	NGS Cecal content	105.5 mg/kg BW	↓ Abundance of Firmicutes and ↑ Bacteroidetes; *↑ Bifidobacterium* and *Lactobacillus*; ↑ *Abundance* of *Lria*, *Akkermansia* and *Lachnospiraceae*; Improvement of structure and villi of the small intestine;
Peng, et al., 2019 [20]	Purified anthocyanin from *L. ruthenicum* (200 mg/kg BW) + DSS	DSS	Oral (water)/7 days	NGS Feces samples	200 mg/kg BW	↑ mRNA of ZO-1, occludin, claudin-1; ↑ total SCFA in cecal content and feces; ↑ goblet cells ; ↑ abundance of Actinobacteria; 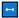 Abundance of *Firmicutes* and *Bacteroidete*; 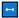 *Firmicutes*/*Bacteroidetes* ratio.
Su, et al., 2019 [21]	Pelargonidin-3-O-glucoside (150 mg/kg BW) from raspberry + Diabetic *db/db*	Diabetc *db/db*	Oral (gavage)/8	NGS Caecal content	150 mg/kg BW ****	↓ Abundance of *Firmicutes* and ↑ *Bacteroidetes*; ↓ serum LPS; ↑ *Bacteroidetes*/*Firmicutes* ratio; ↑ Total SCFA fecal; ↑ mRNA of occludin e ZO-1, Muc 2, and 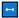 claudin; ↑ Pla2g2 and Lyz1 (antimicrobial peptides).
Tian, et al., 2019 [22]	*L. ruthenicum* dried (3%) + Normal diet	Normal diet	Oral (diet)/10	NGS Fecal pellets	104.2 mg/100 g diet	↓ Abundance of *Firmicutes*; ↓ pH feces; ↓ Serum LPS; ↑ Serum and colon sIgA; ↑ *Verrucomicrobia* and *Bacteroidetes*; ↓ *Proteobacteria* and *Deferribacteres*; ↑ Total fecal SCFA; ↑ Ileal villus length and ratio of villus to crypt; ↑ mRNA of ZO-1, occludin, JAM-A and Muc2; 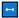 Colon crypt length.
Zary-Sikorska, et al., 2019 [42]	Purple carrot root (dried) (10%)	Control (without carrot)	Oral (diet)/4	NA	12.9 mg/100 g diet	↓ Cecal pH; ↑ α- and β-Glucosidase; α- and β-Galactosidade; β-glucuronidase; ↑ Total cecal SCFA.
Cao, et al., 2020 [30]	Blackcurrant extract (1%) + Old rats	Old rats	Oral (diet)/16	NGS Feces samples	17.41 mg/100 g diet	↓ *Firmicutes*/*Bacteroidetes* ratio; ↓ Abundance of *Verrucomicrobia*, ↑ *Bacteroidetes* and 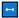 *Firmicutes* and *Proteobacteria*.
Peng, et al., 2020 [24]	Anthocyanins from *L. ruthenicum* (200 mg/kg BW)	Control (without anth.)	Oral (diet)/12	NGS Feces samples	200 mg/kg BW	↑ nº of intestinal villi, goblet cells and intestinal gland; ↑ mRNA of ZO-1, occludin, claudin and Muc1; ↑ total SCFA (cecal content and feces); ↑ *Barnesiella, Alistipes, Eisenbergiella, Coprobacter* and *Odoribacter*; 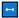 pH in feces and cecal sIgA.
Rodríguez-Daza, et al., 2020 [34]	Blueberry extract (200 mg/kg BW) + High fat and high sucrose diet	High fat and high sucrose diet	Oral (gavage)/8	NGS Feces samples	1.68 mg/kg BW	↑ Mucus layer thickness (colon); ↑ *Adlercreutzia equolifaciens*; 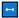 Crypt depth and total goblet cells; 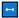 *Firmicutes*/*Bacteroidetes* ratio; 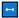 mRNA of ZO-1 and occludin.
Wang, et al., 2020 [25]	Black rice extract (0.48 g/kg diet) + High fat and cholesterol diet	High fat and cholesterol diet	Oral (diet)/12	NGS Caecal content	48 mg/100 g diet	↓ *Firmicutes*/*Bacteroidetes* ratio; ↑ Abundance of *Bifidobacterium* and *Lactobacillus;* ↑ Cecal SCFA; ↑ Villus height (ileum and caecum); ↑ Goblet cell number per villus of the colon; ↑ mRNA of JAM-A, occludin and Muc-2.

↓: reduced; ↑: increased; 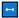: no change; * cyanidin galactoside; ** cyanidin-3-O-glucoside; *** Malvindin 3-Glucoside; **** Pelargonidin-3-O-glucoside; Abbreviations: BW: Body weight; HFD: High fat diet; CPT-11: irinotecan; AOM: azoxymethane; DSS: dextan sodium sulfate; ZO-1: zonula occludentes–1; 3-MCPD: 3-Chloro-1,2-propanediol; SCFA: short chain fatty acids; JAM-A: junctional adhesion molecule-A; *L. ruthenicum*: *Lycium ruthenicum*; CRC: colorectal cancer; Pla2g2: phospholipase A2 group-II; Lyz1: Lysosome-1; LPS: lipopolysaccharides; anth.: anthocyanin; TLR-4: toll like receptor 4; sIgA: secretory Immunoglobulin A; mRNA: messenger ribonucleic acid; Muc: mucin; Defb2: beta-defensin 2; LBS: LPS-binding protein; qPCR: quantitative polymerase chain reaction; CFU: colony forming unit; NA: not applicable; NGS: next generation sequencing; DGGE: denaturation gradient gel electrophoresis.

## Data Availability

The data analyzed in this study are openly available in references number [17,18,19,20,21,22,23,24,25,26,27,28,29,30,31,32,33,34,35,36,37,38,40,41,42,43,44].
